# Family physicians’ moral distress when caring for patients experiencing social inequities: a critical narrative inquiry in primary care

**DOI:** 10.3399/BJGP.2023.0193

**Published:** 2023-11-14

**Authors:** Monica L Molinaro, Katrina Shen, Gina Agarwal, Gabrielle Inglis, Meredith Vanstone

**Affiliations:** Department of Family Medicine, McMaster University, Hamilton, Ontario, Canada.; Department of Family Medicine, McMaster University, Hamilton, Ontario, Canada.; Department of Family Medicine, McMaster University, Hamilton, Ontario, Canada.; Department of Family Medicine, McMaster University, Hamilton, Ontario, Canada.; Department of Family Medicine, McMaster University, Hamilton, Ontario, Canada.

**Keywords:** family practice, health policy, moral distress, primary care, social determinants of health, social welfare

## Abstract

**Background:**

Family physicians (GPs) working with patients experiencing social inequities have witnessed patients’ healthcare needs proliferate. Alongside increased workload demands fostered within current remuneration structures, this has generated concerning reports of family physician attrition and possible experiences of moral distress.

**Aim:**

To explore stories of moral distress shared by family physicians caring for patients experiencing health needs related to social inequities.

**Design and setting:**

A critical narrative inquiry, informed by the analytic lens of moral distress, conducted in Ontario, Canada.

**Method:**

Twenty family physicians were recruited through purposive and snowball sampling via word of mouth and email mailing lists relevant to addictions and mental health care. Physicians participated in two narrative interviews and had the opportunity to review the interview transcripts.

**Results:**

Family physicians’ accounts of moral distress were linked to policies governing physician remuneration, scope of practice, and the availability of social welfare programmes. These structural elements left physicians unable to get patients much needed support and resources.

**Conclusion:**

This study provides evidence that physicians experience moral distress when unable to offer crucial resources to improve the health of patients with complex social needs resulting from structural features of the Canadian health and social welfare system. Further research is needed to critically interrogate how health and social welfare systems around the world can be reformed to improve the health of patients and increase family physicians’ professional quality of life, potentially improving retention.

## Introduction

Family medicine is the dominant primary care specialty in Canada and is a discipline that explicitly encompasses *‘a unique blend of biomedical, behavioural and social sciences, while employing a diverse range of cognitive and procedural skills’*.^1^ Family physicians, elsewhere known as GPs, are integral to this specialty; their care encompasses both health promotion and illness prevention, they coordinate specialty care, and often advocate for patients regarding *‘the care and services they need in all parts of the health care system’*.^1^ What additionally separates family physicians from other health professionals is their focus on complexity and uncertainty, which yields explicit acknowledgment of how social circumstances influence medical issues for many patients.^2^^,^^3^

The relationship between social circumstances and health outcomes is codified in theories of the social determinants of health, which explain how social elements such as housing, income and social protection, social inclusion, and education determine health outcomes.^4^^–^^6^ This allows for an understanding of equity, which is: *‘the absence of unfair, avoidable or remediable differences among groups of people, whether those groups are defined socially, economically, demographically, or geographically or by other dimensions of inequality (e.g. sex, gender, ethnicity, disability, or sexual orientation)’*.^7^

Health equity, thus, is *‘achieved when everyone can attain their full potential for health and well-being’*.^7^ The social determinants of health provide an explanation of how social and health inequity are related, wherein lack of access to social goods can foster negative health outcomes. For example, access to population-based cancer screening is lower for those who experience racism, poverty, or precarious employment, resulting in later diagnoses of cancer and worse health outcomes.^8^^–^^11^

Even though family medicine has a mandate to address social determinants of health and the health inequities stemming from lack of access to social goods,^12^^,^^13^ it is situated within health and social care systems that are ill-equipped to respond to the structural causes of ill health. While the sequelae of social and economic deprivation may not ideally be addressed in a family physician’s office, current social welfare systems are designed such that people who are struggling with addiction, mental ill health, precarious housing, or insufficient income seek downstream solutions (for example, safe drug supply or physician’s signature on social assistance supports) for the systemic conditions in which they are embedded. Primary care represents one of the only consistent, accessible, and publicly funded services available to people with multiple constellations of need, even when medical solutions may not be sufficient to address social and structural challenges.^14^ For instance, while family physicians could prescribe medication for insomnia, this treatment is not able to fully address the challenges of sleeping rough on the street or in crowded shelters, the anxiety of struggling to make ends meet financially, or the chronic stress of social exclusion from being unhoused. This entwinement of social and medical need is particularly acute for marginalised people, including individuals who face racism, poverty, ableism, and dangerous or precarious working conditions.^15^^–^^18^

**Table:** How this fits in

The moral distress of physicians who cannot provide adequate care as a result of systemic deficits is seldom heard in contemporary discussions about healthcare access and quality. Family physicians’ stories of moral distress in relation to structural and systemic factors such as racism, colonialism, and drug, mental health, and housing policy, generate seemingly novel and vital understanding of the clinical work of primary care providers. The findings of this study are some of the first to illustrate family physicians’ experiences of moral distress, contributing to the limited body of literature on moral distress in primary care.

While family physicians working with marginalised patients cannot address the root causes of this insufficient resourcing, they are often left to pick up the pieces of these broader policy decisions when caring for patients. In doing this work, they are aware that patients are experiencing multiple forms of harm and seeking care in a system that is ill-equipped to respond to the root causes of ill health. While bearing witness to the limits of the system, physicians may be unable to offer patients interventions that are most greatly needed. Thus, examining the experiences of family physicians who work with disadvantaged patients is particularly important, as when they are not able to help patients with unmet social needs that result in medical needs there is potential that the physicians may experience moral distress.^19^^–^^21^

Moral distress, defined as *‘when one knows the right thing to do, but institutional constraints make it nearly impossible to pursue the right course of action’*,^22^ has become an increasingly topical concept in scholarly literature and news media as a significant issue facing healthcare providers worldwide.^23^^–^^33^ However, moral distress has rarely been examined in the context of family medicine, and even less so in relation to the experiences of family physicians who specifically work with patients experiencing health needs related to social inequity.

Examining moral distress in this context is timely, as the discipline of family medicine has recently garnered attention for the increasing and disconcerting shortages of family physicians internationally.^34^^–^^42^ According to physicians who have left family medicine, the accumulation of conflicting, simultaneous increases in clinical care responsibilities and increasingly complex health needs of patients has been the impetus for leaving.^34^^–^^42^ What has not been explored is whether these experiences are a result of, or possibly underpinned by, moral distress. Thus, the purpose of this study was to explore family physicians’ stories of moral distress in caring for patients experiencing health needs related to social inequities.

## Method

### Study design

This is a critical narrative inquiry informed by Jameton’s conceptualisation of moral distress.^22^ Critical narrative approaches are paradigmatically bound in critical theory, which aims to challenge and disrupt the status quo, with the ultimate goal of transformation.^43^^,^^44^ When critically examining narratives, one can understand how a person’s morality is both embedded within how they narrate their connections to others, and *‘within issues of power and control’*.^45^ When a person’s sense of identity is challenged or threatened, stories are useful for making sense of the ambiguities that characterise the person’s relationships with others and with the structures within which they are embedded.^46^^–^^48^ By critically interrogating these broader structures, narrative research can drive structural and institutional change. Stories also have persuasive value, particularly in policy decision-making contexts where decision makers are tasked with making sense of multiple (and conflicting) forms of evidence.^49^ Additional information regarding the methodology used for this study can be found elsewhere.^50^

### Participant recruitment

Family physicians who were practising in Ontario, Canada, and who self-identified as working with patients experiencing health needs related to social inequity (such as inadequate housing and issues with employment, income, or transportation) were eligible to participate. Ontario, Canada, in addition to being where the authors work, is an integral location for understanding experiences of working with patients who experience inequities. Similar to many other provinces, states, and countries internationally, Ontario has a large population where wealth is generally concentrated in urban pockets, often leaving rural regions medically underserved.^51^^–^^53^

All participants were recruited via purposive and snowball sampling. First, study collaborators emailed study materials to potential participants in their networks who worked with patients experiencing inequities. Study materials were also circulated via an email list of physicians working with patients who use substances. Any potential participants were encouraged to consider the social inequities their patient groups experience — the researchers did not suggest forms of inequity that these patients may experience.

The intent of narrative inquiry is not to generalise findings to a particular population, but to generate rich data concerning a particular experience.^54^^,^^55^ In particular, judgements of data sufficiency in narratological research focus on assessing the depth of participants’ stories and not on the quantity of possible participants. As such, the goal was not to recruit a large number of participants and reach saturation in the data, but rather to recruit a small number of participants who could speak at length about their work with marginalised patients. In this way, their interviews would generate rich stories that may be transferable to other family physicians and health professionals working with a similar population of patients.

### Data collection

Consistent with critical narrative approaches,^56^ participants were invited to two narrative interviews with one researcher that were conducted over the phone or via an online video meeting. The first interview acted as an opportunity for participants to freely narrate their experiences working with patients experiencing inequities, with the researcher probing in response to their stories (see Supplementary Box S1 for details). The second interview was an opportunity to probe for more *‘narrative detail’ *^56^ that may have been missed in the original interview, such as the relevance of particular remuneration structures. The determination of relevance arose from the analysis of the first interview data. Stories of moral distress were told in response to specific interview questions and spontaneously in conversations about what it meant to provide care for patients experiencing social inequity. These interviews were transcribed, analysed, and sent to participants for review. To uphold trustworthiness and sincerity, an audit trail was maintained that included field notes, decisions regarding methods, feedback from collaborators to strengthen interpretations or future research decisions, and reflexive statements.^57^^,^^58^

### Data analysis

Data analysis methods were consistent with critical narrative approaches.^55^^,^^59^ Narrative inquiry does not aim to uncover an objective truth among participant stories that is generalisable, correlational, and comparable across populations.^60^ Rather, rigorous narrative research can be recognised by an analysis that inspires reflection by *‘prompt*[ing] *readers to think beyond the surface of a text’*^61^ and consider the broader contexts that the narrative is situated within. Narrative analyses are iterative, and while grounded in the data, are based on the interpretations of those analysing the data. In the case of this particular study, the researcher followed the approaches of Lieblich *et al*^55^ and Rudman and Aldrich,^59^ whose analysis approaches consist of conducting multiple close reads of the data until a pattern is identified; noting any ambivalences, contradictions, or tensions to the pattern; deciding on and highlighting key content or themes; reading the data for each identified theme; and noting the distinctive features (that is, context and transitions) of each theme. Many of the narrative themes were independently substantiated by another researcher via a separate analysis of all transcripts. These interpretations were presented to the remaining three researchers, whose experience as clinicians and as family medicine researchers helped confirm that interpretations were grounded in the data, and provided assistance in refining and contextualising the interpretations.

## Results

In total, 20 family physicians working throughout Ontario (Table 1) participated in 36 narrative interviews (four participants were lost to follow up after their first interview). While not all participants explicitly expressed the payment model they work within, many drew on their experiences working in different payment models. These payment models included fee-for-service (billing the provincial health insurance plan for reimbursement for each medical service provided), salaried, and blended capitation (a payment per patient enrolled, regardless of service use paired with fee-for-service elements) models.

**Table 1.** Demographics of participants, including work location, practice structure, and areas of focused practice

**Variable**	***n***
**Sex**	
Male	7
Female	13

**Age, years**	
25–29	1
30–34	4
35–39	3
40–44	4
45–49	1
50–54	2
55–59	4
60–64	1

**Work location**	
Urban	12
Rural	3
Both	5

**Practice structure^a^**	
Family health team	7
Family health organisation	1
Community health centre	3
Indigenous primary healthcare organisations	2
Independent solo	2
Independent group	3
Locum	3
Outpatient	2
Hospitalist	2

**Areas of focused practice^b^**	
Addiction medicine	4
Mental health	5
Unhoused patient care	4
Incarceration care	3

**Years of experience**	
>1–4	6
5–9	3
10–14	3
15–19	1
20–24	0
25–29	5
30–34	0
35–39	2

a *Family practice structures total more than 20 as some participants worked in more than one type of practice structure.*

b *Areas of focused practices where there were any.*

Participants described moral distress as a ubiquitous feature of their professional lives; their narratives suggested they were often constrained in their ability to decide on and do what they felt was right for patients. For example:

*‘Powerless* [to] *help them* [patients] *reach any kind of wellbeing in life if they’re still struggling with just putting a roof over their heads. There’s no amount of medication that I’m going to prescribe that’s going to make that okay*.*’*

(Primary care provider [PCP]120)

This description of powerlessness to help with available resources was echoed widely throughout the dataset through descriptions of feeling *‘helpless’* (PCP103, PCP106, PCP109, and PCP112), and *‘hopeless’* (PCP106, PCP111, and PCP112) about what could actually be done to help patients. Others’ moral distress was elucidated through their descriptions of work being *‘soul-sucking’* (PCP111), *‘demoralising’* (PCP102), and *‘frustrating’* (PCP105, PCP107, and PCP113).

Below, representative quotes are presented from two narrative themes that were generated from, and illustrate the roots of, physicians’ moral distress. These narrative themes address how policies governing physician remuneration and scope of practice left participants feeling overwhelmed with demands and forced to make compromises in what they chose to address; and how participants were limited by the behaviours of some patients, whose behaviours were influenced by and embedded within broader systemic constraints and availability of social welfare programmes, leaving physicians unable to get them the resources needed to survive, let alone thrive.

### *‘A death of 1000 cuts over time’*: increasing workload demands on already high workloads

A baseline contributor to experiences of moral distress, as narrated by the participants, was the increasing workload responsibilities taken on because of new family medicine remuneration and scope of practice structuring, leaving them with even less time to care for complex patients. This was exemplified by one participant (PCP120), who described how, while family physicians are *‘pretty adaptive people’*, the *‘downloading* [of] *more work on to family doctors’*, and the *‘increasing requirements and demands from multiple sources’* signalled a continuous *‘eroding’* of his (and his colleagues’) identity and values. His story suggested that he and other family physicians are now *‘negotiating’* between completing remunerable tasks or providing unremunerated, dignified care for socially complex patients who need more than a typical 15-minute visit:

*‘But when there’s just constantly more, being added to the expectations, that’s very, very demoralising for sure. Because then, remember, it’s, it’s not just a single negotiation of our identity and values and moral distress, it’s a continuous one where it’s continually eroding and becomes a death of 1000 cuts over time.’*

(PCP120)

This description of moral distress as a *‘death of 1000 cuts over time’* exemplified the slow but continuous breakdown of the physicians’ moral resolve in their care giving, and drew attention to an identity tension of being the physician they wanted to be and the physician they had the time and resources to be. Demonstrating this erosion of values in having to choose between workload and remuneration demands and dignified patient care, one participant (PCP113) narrated her own death of 1000 cuts by admitting that, while she *‘love*[s] *what I do on some days’*, if she were *‘back in medical school right now, knowing what* [she now] *know*[s]*’* she would not have chosen to be a family doctor due to what the workload is like:

*‘I just think the risk of burnout, and the amount that’s being asked of family doctors in our province, versus the compensation, and the ability to actually balance work– life, if you’re truly trying to live up to the aspirational goals of what a primary care provider should be, are just too great, right? Like, why would anybody want to do my job when they could work less hard, be happier, have more time with their families, have more time for their own wellbeing?’*

(PCP113)

Her narrative further suggests that family physicians in Ontario are undervalued, and the realities of practice conflict with the values that she assigns to being a family physician and providing optimal care for patients. This conflict, and constraint in being able to perform this care, caused her moral distress.

### *‘They’re called “heart-sink” patients because when I see their names my heart sinks’*: personal, structural, and systemic barriers

Some physicians’ narratives suggested that their moral distress stemmed from patients’ engagement in behaviours that ultimately limited their ability to receive care. This was exemplified by one participant (PCP110), who drew comparison between *‘systems issues’* and *‘individual issues’* in her story of *‘a young man that wants to start on an opiate replacement therapy’*. She believes his initiative is *‘wonderful’*, but knows *‘his behaviour has been so bad that there’s not a pharmacy that will accept him, because he’d been so abusive, or he’s stolen so many times’*:

*‘I can’t ask the pharmacist to give him one more chance when the first 13 chances didn’t go well … I have nothing to offer him … He’s probably going to die, he’s probably going to overdose … I have nothing to offer. There’s nothing anybody can offer him … It was his own behaviours, and unfortunately, he’s generally not a nice person … That doesn’t mean I won’t see him, but it can sometimes really limit — it’s not just … there’s nothing I can offer him*.*’*

(PCP110)

Her narration also draws attention to her feelings of moral distress and continued empathy through her repeated use of the phrase *‘there’s nothing I can offer him’*. This draws attention to the moral constraint she feels as a result of the patient’s behaviours, knowing that broader structures had bearing on his issues with his mental health and addictions, and ultimately shaped his current behaviour.

Many of the physicians’ stories highlight how their moral distress emanates from these *‘systems problems’* (PCP110), conveying that *‘institutionalised obstacle*[s] *or hardships that the patient experiences in society, not even just in health care’* (PCP102), particularly social welfare systems, have undeviating effects on patients’ lives. However, addressing the issues generated by these poorly structured social welfare systems are not within their ability to help make meaningful change for patients. One participant (PCP105), for instance, drew attention to her feelings of constraint by describing the *‘huge gap’* between what her patients’ *‘needs are and what I can actually offer for them’*:

*‘Here I am saying to somebody, “well, you need to take this medication because it’s gonna help with your depression” … and really all they’re thinking is, “Right now I just don’t wanna leave your office because it’s warm, and I don’t have a place to live. So can I hang out here a little bit longer and, you know, maybe have a cup of coffee?”.’*

(PCP105)

Some participants expressed this sentiment more explicitly, such as one (PCP101), who, when asked if she experienced moral distress in her work, immediately responded with *‘Yes, totally. Oh my goodness’*, and related her experience to structural factors of her patients’ lives that she does not have the power to alleviate:

*‘Yeah, lots of the patients I care for die young because of our shitty toxic drug policy, and because of colonisation, and because of racism. Uhh, yeah, that’s totally morally distressing.’*

(PCP101)

From there, she compared her experiences to those of her patients who live in these conditions, by stating that she does not *‘think that I experience a ton more moral distress as a physician than … people experience living in their own communities that are under these terrible conditions of violence’*. In this narration, she minimises her own experiences of moral distress in relation to the trauma her patients have experienced, and continues to state that *‘it’s probably better to experience moral distress and acknowledge it and work through it’* than become unattached from her patients through *‘moral disintegration or moral detachment or, or you know other ways of dealing with those negative … emotions that come with moral distress’* (PCP101). Through this narration, she suggests that remaining attached to and caring for patients is more important than making changes to remediate her own moral distress.

## Discussion

### Summary

The stories of 20 family physicians who care for patients with medical needs related to social inequity highlight how moral distress can be grounded in broader systemic and structural factors. The study findings, to the authors’ knowledge, are some of the first to illustrate the moral distress experienced by family physicians, particularly those working with patients experiencing inequities, which builds on the small body of moral distress scholarship in the primary care literature.^62^^–^^68^ Participants’ moral distress was associated with remuneration structures and workload demands, and was exacerbated by a lack of social resources, such as housing, and insufficient comprehensive mental health and addictions services, which resulted in strict limitations on their ability to provide comprehensive care.

### Strengths and limitations

A strength of this study is consistency in its focus on narrative. Through the conceptualisation of the research, narrative-eliciting interviewing approach, and highly immersive, multi-stage interpretive process, the study was epistemologically and methodologically tethered, which ensured rich data and substantial depth to the analysis. Further, the use of a critical narrative methodology allowed for a nuanced understanding and situating of participant narratives in broader institutional and structural contexts.

A considerable strength of this research is its transferability to other provinces, countries, and healthcare contexts where the social safety net is weak or non-existent. In these cases, the downstream effects of limited social support are often brought to family physicians, who are insufficiently resourced to provide care for the upstream determinants of health.

The research is bound by the historical and geographical contexts in which it was conducted. While the findings are not intended to be generalisable to all family physicians, they may have less salience with family physicians working with patient populations who are not marginalised. Additionally, in contexts where social welfare policy and the social safety net are strong, such as countries in which safe and affordable housing and safe drug supply are available, these experiences may not be as relevant.

### Comparison with existing literature

The stories told by participants in this study are congruent with the growing attention to the attrition of family physicians in many countries.^69^^–^^71^ Issues such as increasing workload demands related to escalating indirect care requirements, and the increased complexity of patients’ health needs because of delayed screenings during the COVID-19 pandemic have previously been cited as reasons for the mass exodus of clinicians out of family medicine.^34^^–^^42^ The findings of the current study are also congruent with research on American family medicine residents that demonstrates how workload and limited time to care for oneself contributes to moral injury, which is related to but distinct from moral distress.^72^ Moral injury is regarded as a deep violation of what one believes is right, and can be the identity- changing result of experiencing multiple morally distressing events.^73^ The findings from this work on residents draws attention to the fact that family physicians may be experiencing moral distress from their clinical work as early as their residency,^72^ drawing attention to the broader structuring of health and social care systems that physicians are embedded within. As long as upstream social welfare policy issues remain unresolved, family physicians will continue to compensate for them, maintaining the likelihood that they will continue to experience moral distress.

There are also similarities between the findings of the current study and reports of American clinicians’ moral distress during the COVID-19 pandemic.^67^ These clinicians also experienced moral distress related to patients not receiving the best or necessary care, and bearing witness to the inequities experienced by patients, including homelessness and racism.^67^ It should be noted that the participants in the current study did not experience moral distress by proxy of working with persons experiencing inequities. Rather, similarly to the aforementioned study,^67^ moral distress was experienced by being rendered unable to help patients whose issues would be alleviated by stronger social welfare policy, and being embedded within both social welfare and healthcare systems that are ultimately harming patients.

### Implications for research and practice

Participants’ stories of moral distress in relation to structural and systemic factors provide a novel illustration of how a lack of social welfare support results in compounding health needs brought to family physicians, who may not have access to corresponding resources.^74^^,^^75^ This is also exemplified through the links drawn between individual behaviours and systems issues. While some physicians may see patient choices as non-compliance, the participants’ stories in this study illustrate how the choices patients make are influenced, in part, by the systems they are bound within. As an example, a patient who takes their daily medication every second day and delays renewing their prescription for months may be perceived by some as non-compliant with treatment protocols. However, when contextualised with information about the patient’s low disability support payments and lack of pharmaceutical insurance coverage, these behaviours can be seen as directly related to structural causes of poverty.

This study has implications for primary care policy and practice, particularly through invocations to restructure family physician remuneration to encourage sensitive work with complex patients. The findings illustrate that remuneration is an important factor in what care, if any, marginalised patients can receive. Capitation or salaried models, while not a perfect solution to blended fee-for-service models,^76^^–^^78^ may offer physicians more time and ability to care holistically for patients with complex needs. Additionally, the use of interprofessional teams, in which patients have free access to professionals such as social workers, legal advisers, and system navigators may better address patient needs.^41^^,^^79^^,^^80^

While this would allow for better care for marginalised patients, these changes will not ultimately address the upstream deficiencies in social welfare systems that deprive people of safe and affordable housing, consistent and well-paying employment, and safe drug supply. The social and structural challenges faced by people experiencing inequity cannot be resolved until the prioritisation of consistent, accessible, publicly funded social services takes precedence in social and political agendas worldwide.

Future research should examine experiences of moral distress in all forms of integrated care that constitute family medicine and general practice, in order to understand the links between moral distress and stringent primary care budgeting in differing institutional contexts. Further, while current remedies to moral distress include suggestions of practising wellness and resilience,^81^^–^^83^ these interventions do not include focus on the structural and systemic roots of moral distress. Future research should therefore examine what bearing institutional and structural change has on experiences of moral distress.

## Data

The dataset relied on in this article is available from the corresponding author on reasonable request.
